# Attention Improves During Physical Exercise in Individuals With ADHD

**DOI:** 10.3389/fpsyg.2018.02747

**Published:** 2019-01-09

**Authors:** Yuri Rassovsky, Tali Alfassi

**Affiliations:** ^1^Department of Psychology, Bar-Ilan University, Ramat-Gan, Israel; ^2^The Leslie and Susan Gonda (Goldschmied) Multidisciplinary Brain Research Center, Bar-Ilan University, Ramat-Gan, Israel; ^3^Department of Psychiatry and Biobehavioral Sciences, University of California, Los Angeles, Los Angeles, CA, United States

**Keywords:** physical exercise, attention, ADHD, continuous performance test, reaction time

## Abstract

The present study examined the effects of physical exercise on attentional processes in individuals diagnosed with Attention Deficit Hyperactivity Disorder (ADHD), compared to healthy controls. Unlike previous studies typically comparing performance on baseline measures with post-exercise performance, this study examined the effects of physical exercise on attention while participants were engaged in a continuous performance task. Fourteen individuals diagnosed with ADHD (71% females, mean age = 24.8) and 17 controls (76% females, mean age = 22.6) completed the Conners Continuous Auditory Test of Attention (CATA). All participants completed the test twice, at baseline in a sitting position and while walking on the treadmill at a speed of 5 km/h. The order of administration was counterbalanced for each group. A 2 × 2 ANOVA with repeated measures detected a group by activity interaction on several measures of the CATA. Specifically, compared to baseline, the ADHD group demonstrated faster reaction times during physical exercise (25.4 ms faster) and decreased omission errors (1.5% better), whereas controls showed the opposite pattern (15.9 ms slower and 0.88% worse, respectively). Importantly, the ADHD group’s overall relatively lower performance on these measures was only evident in the resting condition, attaining scores similar to controls during exercise. These results suggest a possibly hypoactive attentional system in ADHD that could potentially be enhanced by arousal through engagement in physical exercise.

## Introduction

Attention-Deficit/Hyperactivity Disorder (ADHD) is characterized by difficulties paying and/or sustaining attention, often accompanied by deficits in controlling or inhibiting behavior (American Psychiatric Association, 2013). In efforts to explain the variability in the different and complex problems exhibited by individuals with ADHD, contemporary leading theories have identified the core of the pathology with a deficit in attentional and executive functions, such as sustained and divided attention, attentional and inhibitory control, working memory, planning, cognitive flexibility, and problem solving (Petersen and Posner, 2012; Diamond, 2013). Neuropsychological assessments of these deficits typically include, among others, continuous performance tasks (CPTs), which assess the various aspects of these attentional processes. Studies employing these measures report a clear pattern of performance distinguishing adults with ADHD and healthy adults, such that ADHD participants demonstrate significantly more omission and commission errors and lower sensitivity (Riccio and Reynolds, 2001).

Accumulating data suggest a positive effect of physical activity on cognitive functioning in ADHD. These positive effects are supported both by animal and human studies. Robinson et al. (2011), for example, studied spontaneously hyperactive adolescent rats and found that engagement in physical exercise resulted in subjects’ reduced distractibility and improved attentional capabilities. Studies in humans reported similar findings. Pontifex et al. (2013), for example, showed that both children with ADHD and a matched, healthy control group exhibited improved cognitive performance on measures of executive and attentional functions, as well as reduced ADHD symptoms, following moderate physical activity. Gapin and Etnier (2010) found that more frequent physical activity was associated with better executive functioning, including inhibition, planning, working memory, and processing speed, in children with ADHD. Chang et al. (2012) found that moderate to intense aerobic exercise facilitated inhibition and set shifting, functions that characterize the primary executive deficits in ADHD.

Most prior studies examined the effects of physical activity on attention by assessing attentional performance before and immediately following physical training Lambourne and Tomporowski (2010). To expand on this literature, the present pilot study aimed to examine the influence of physical exercise conducted during performance of an attentional task. To this end, individuals with ADHD and a normative control sample engaged in physical activity, while simultaneously performing an auditory CPT. We capitalized on previous efforts to explain the mechanism underlying ADHD-related deficits as an imbalance in the catecholaminergic systems in the brain, leading to dysfunction of attentional circuits (Yu-Kai et al., 2012). Specifically, it has been hypothesized that attentional impairments may be due to a hypodopaminergic state in the prefrontal cortex, as supported by the common use of stimulant medications in the treatment of ADHD (Solanto, 2002). Therefore, given the potentially lower arousal levels among individuals with ADHD, we predicted a greater benefit to emerge among individuals with ADHD, as compared to healthy controls.

## Materials and Methods

### Participants

Participants were 31 physically healthy Bar-Ilan University students (74.2% women), who were recruited through ads placed around the university campus and on the internet. The sample’s average age was 24.13 (*SD* = 4.27, range 18–42), and their average years of education was 15.6 (*SD* = 2.15, range 12–19). Fourteen participants had a formal diagnosis of ADHD, given by their neurologist or psychiatrist, and 17 participants served as controls, with no history of psychiatric or neurological diagnoses. Mean age of ADHD diagnosis was 15.7 (*SD* = 4.39), with 42% hyperactive type. Most ADHD participants (71%) had prescriptions for a stimulant medication (Ritalin, Concerta, or Adderall). Upon arriving at the lab, participants received a full explanation of the research procedures from a trained research assistant. All participants then gave a written informed consent according to procedures approved by the Bar-Ilan University Institutional Review Board.

### Measures and Procedure

All participants completed the Conners Continuous Auditory Test of Attention (CATA), administered on a Dell Pentium computer, driven at 160 Hz. The CATA is a standardized continuous performance test, with negligible practice effects (Conners, 2014). It is administered in the auditory modality through earphones, thereby minimizing distractibility that may be difficult to overcome when exercising using visual tasks. The CATA task assesses auditory processing and attention-related problems in individuals aged 8 years and older. The task has 200 scored trials, divided into 4 blocks of 50 trials. Within each block, 80% of the trials are warned trials, on which a low tone (warning) is followed by a high tone (target). The remaining 20% of the trials are unwarned, in which the high tone is played without warning (non-target). Participants are asked to respond by left-clicking the mouse with the index finger of their dominant hand as fast as they can to high tones on warned trials but ignore high tones on unwarned trials.

The measures used in the present analyses are described in detail elsewhere (Kallo and Rassovsky, 2017). Below is a summary of these measures:

(1) Detectability (DPR) is a measure of the respondent’s ability to discriminate targets (i.e., the high-tone sound on warned trials) from non-targets (i.e., the high-tone sound on unwarned trials), with higher raw and T score value indicating worse performance (i.e., poorer discrimination).
(2) Hit Reaction Time (HRT) is the mean response speed, measured in milliseconds, for all non-perseverative target responses made during the entire administration.
(3) Omissions (OMI) are missed targets, indexed by the percent of non-responses to targets.
(4) Commissions (COM) are incorrect responses to non-targets, indexed by the percent of responses to non-targets.
(5) C is a signal detection statistic that measures an individual’s natural response style and can be classified as having a conservative style that emphasizes accuracy over speed, a liberal style that emphasizes speed over accuracy, or a balanced style that is biased neither toward speed nor toward accuracy. Higher T scores represent more conservative response style.

The test was administered twice, at baseline in a sitting position and while walking on a York treadmill (model Capital25) at a speed of 5 km/h, both closely supervised by a trained research assistant. Given the moderate intensity of this activity, no “warm-up” period was included, and the entire duration of the exercise was 14 min, which is the time needed to complete the CATA. The order of administration was counterbalanced for each group. Participants with ADHD were instructed to refrain from using medications on the day of the experiment, and all participants were instructed to refrain from drinking coffee or high-sodium drinks.

### Data Analysis

Due to significant age difference between the ADHD and control groups, exploratory analyses for identification of outliers were conducted for age-matching. These analyses identified one 42-year-old participant in the ADHD sample, who was excluded from subsequent analyses. Potential age and gender differences between the groups were examined using independent samples and Chi-square test, respectively. A 2 × 2 analysis of variance (ANOVA) with repeated measures was used to investigate the effects of exercise on attention, followed by *post-hoc* analyses with Bonferroni correction to decompose the two-way interactions. Exercise was the within-subjects variable and ADHD diagnosis was the between-subjects variable. All the aforementioned measures generated by the CATA were converted to T-scores and entered as dependent variables.

## Results

Independent samples s detected no significant age difference between the ADHD (*M* = 24.8, *SD* = 3.49) and control (*M* = 22.6, *SD* = 1.54) groups, *t*(28) = 2.10, *ns*. No significant differences were furthermore found in the distribution of males and females in each group (*χ^2^*= 0.001, *df* = 1, *ns*). The procedure was well tolerated by all participants, with no dropouts.

A 2 × 2 ANOVA with repeated measures was conducted for each of the key dependent variables of the CATA, followed by *post-hoc* analyses with Bonferroni correction to decompose the significant two-way interactions. The descriptive and inferential statistics are presented in Table 1. As can be seen in Table 1, a significant main effect of Activity was detected for DPR (*p* = 0.007, $\eta_{p}^{2}$ = 0.235) and COM (*p* = 0.001, $\eta_{p}^{2}$ = 0.348), indicating that both groups demonstrated lower ability to discriminate targets from non-targets and made more commission errors (incorrect responses to non-targets) in the exercise than in the sitting conditions. None of the other main effects of Group or Activity reached statistical significance.

**Table 1:** Performance data on measures of attention.

					Main effect of	Main effect of	Interaction
	ADHD *n* = 17		Controls *n* = 14		group *F*	activity *F*	effect *F*
	Baseline *M* (*SD*)	Exercise *M* (*SD*)	Baseline *M* (*SD*)	Exercise *M* (*SD*)			
DPR	51.23 (9.33)	53.39 (5.41)	48.65 (5.46)	53.65 (7.69)	0.252	**8**.**61**	1.363
HRT	373.1 (102.3)	347.7 (58.3)	325.3 (51.9)	341.2 (68.8)	1.19	0.308	**5**.**80**
OMI	48.08 (5.35)	46.62 (2.18)	45.53 (.875)	46.41 (1.58)	2.27	0.319	**5**.**22**
COM	49.08 (4.03)	52.08 (6.68)	47.82 (3.99)	51.18 (5.94)	0.377	**14**.**9**	0.046
C	48.77 (3.68)	46.39 (4.66)	47.18 (8.05)	47.47 (7.53)	0.013	1.43	2.34


*DPR, detectability; HRT, hit reaction time; OMI, omissions; COM, commissions; C, response style. *F* values in bold indicate significance at *p* < 0.05 level.*

**FIGURE 1:**
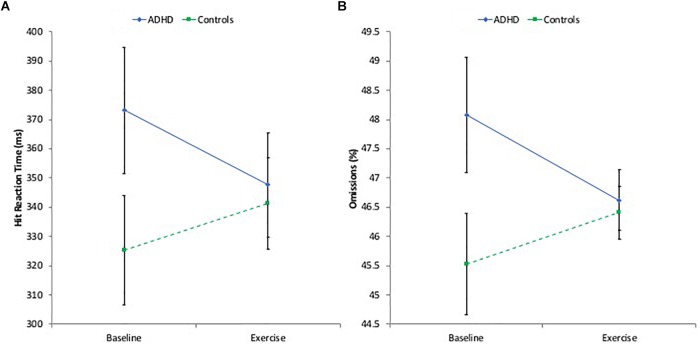
**(A)** CATA hit reaction time (higher ms reflect poorer performance); **(B)** CATA omission errors (higher % reflect poorer performance).

Analyses furthermore revealed a significant Group by Activity interaction for HRT (*p* = 0.02, $\eta_{p}^{2}$ = 0.172), such that the ADHD group showed decreased reaction time (faster response) during the exercise condition, whereas the control group showed increased reaction time (slower response) during the exercise condition (see Figure 1A). *Post-hoc* analyses with Bonferroni correction for multiple comparisons to decompose the significant two-way interaction indicated a substantially larger change (25.4 ms decrease in HRT from baseline; *p* = 0.06, $\eta_{p}^{2}$ = 0.122) in the ADHD group than in controls (15.9 ms increase in HRT from baseline; *p* = 0.17, $\eta_{p}^{2}$ = 0.066). Similarly, there was a significant Group by Activity interaction for OMI (*p* = 0.03, $\eta_{p}^{2}$ = 0.157), such that the ADHD group made less omission errors during the exercise than in the sitting condition, whereas the opposite pattern was seen in controls (see Figure 1B). Again, *post-hoc* analyses with Bonferroni correction indicated larger change (1.46% decrease in omission errors; *p* = 0.07, $\eta_{p}^{2}$ = 0.113) in the ADHD group than in controls (0.88% increase in omission errors; *p* = 0.20, $\eta_{p}^{2}$ = 0.057).

## Discussion

The present pilot study examined the effects of physical exercise on attention in adults diagnosed with ADHD, as compared with a normative control group. Unlike most prior studies, participants completed a CPT measuring sustained attention while engaging in physical exercise. Analyses revealed significant group by activity interactions, such that, compared to healthy controls, participants with ADHD demonstrated faster reaction times (HRT) and less omission errors (OMI) while walking, as compared to sitting, and their relatively lower performance on these measures was only evident in the resting condition, attaining scores similar to controls during exercise.

These findings are in line with accumulating evidence suggesting the benefits of participating in physical exercise programs for adults with ADHD (Archer and Kostrzewa, 2012). Importantly, Kessler et al. (2010) reported that about half of their adult subjects diagnosed with ADHD in childhood showed symptoms compatible with current adulthood ADHD diagnosis. It appears then that a substantial number of children suffering from ADHD continue to have attentional difficulties as adults. Silva et al. (2015) indicate that, after engaging in physical exercise, children with ADHD may have comparable concentration abilities to children without ADHD. Based on these findings, Hill et al. (2011) implemented an exercise intervention program in schools, demonstrating beneficial outcomes of classroom-based exercise regime. These data suggest, therefore, that it could be beneficial to introduce a physical exercise regimen to the ever-increasing sedentary adult lifestyle, with an added benefit for attentional functions among adults with ADHD.

Several potential mechanisms have been proposed to underlie the beneficial effects of exercise in ADHD. Given the increased release of catecholamines during physical activity, Wigal et al. (2012) suggested that the beneficial effects of exercise on attention might be mediated through the same catecholaminergic systems targeted by stimulant medications for ADHD. Similarly, Chang et al. (2012) suggested that the positive effects of physical exercise on executive functions might be due to induced dopamine release. They offer two additional processes that could contribute to the positive exercise effects. First, they suggest that acute exercise may be accompanied by activation in the dorsolateral prefrontal cortex, an area of the brain that has been implicated as dysfunctional in ADHD. They furthermore suggest that acute exercise improve inhibition and may thus be accompanied by changes in attentional resource allocation, resulting in increased stimulus evaluation and recognition. Given the potential engagement of attentional and executive systems through exercise, interventions focused on physical activity might be effective in managing the disorder’s core symptoms (Cerrillo-Urbina et al., 2015).

In contrast to some prior studies (e.g., Pontifex et al., 2013), the control group in our study did not benefit from simultaneous cognitive and physical engagement, and in fact experienced decline in performance on some measures. It is possible that, unlike the under-engaged attentional processes in ADHD, due to hypoactive catecholaminergic system (Yu-Kai et al., 2012), attentional mechanisms in the control sample tend to be nearly optimized at rest. This interference in the performance among controls in the exercise condition might have therefore resulted from the cost of performing a dual task (engaging in physical exercise in parallel to executing the CATA).

The present study was an initial pilot to examine performance on CPT conducted concurrently with physical exercise, and its conclusions are limited by the relatively small sample size. Another limitation of this study is that all participants exercised at the same intensity level, without controlling for several important physiological parameters, such as aerobic fitness, heart rate, and BMI, which could have moderated individual responses. Nonetheless, significant interaction effects, with moderate effect sizes, were found on several key measures, suggesting a potential benefit of physical activity to attentional processes in adult ADHD. It adds to the growing body of research supporting the beneficial effects of exercise on cognitive functions. Together, these studies suggest that, in addition to the myriad of physical and cognitive benefits that can be derived from regular exercises, physical activity while performing attentional tasks may bolster cognitive performance in individuals with ADHD. Upon replication in larger samples, these findings could be readily translated to practical applications in the classroom or working environment. Pedaling on a stationary bicycle or walking on a treadmill during lecture or work may prove beneficial for both physical and brain health.

## Ethics Statement

This study was carried out in accordance with the recommendations of APA ethical guidelines, as approved by Bar-Ilan University Institutional Review Board. All subjects gave written informed consent in accordance with the Declaration of Helsinki.

## Author Contributions

All authors have contributed significantly to the manuscript. YR conceptualized the study, analyzed the data, and wrote the manuscript. TA assisted in study conceptualization, data analyses and manuscript preparation, and collected the data.

## Conflict of Interest Statement

The authors declare that the research was conducted in the absence of any commercial or financial relationships that could be construed as a potential conflict of interest.
